# Usefulness of quantitative ^99m^Tc-pyrophosphate SPECT/CT for predicting the prognosis of patients with wild-type transthyretin cardiac amyloidosis

**DOI:** 10.1007/s11604-021-01221-6

**Published:** 2022-01-01

**Authors:** Kouji Ogasawara, Shinya Shiraishi, Noriko Tsuda, Fumi Sakamoto, Seitarou Oda, Seiji Takashio, Kenichi Tsujita, Toshinori Hirai

**Affiliations:** 1 Department of Diagnostic Radiology, Graduate School of Life Sciences, Kumamoto University, 1-1-1 Honjo, Chuo-ku, Kumamoto City, 860-8556 Japan; 2 Department of Diagnostic Medical Imaging, School of Health Faculty of Life Sciences, Kumamoto University, 1-1-1 Honjo, Chuo-ku, Kumamoto City, 860-8556 Japan; 3 Department of Cardiovascular Medicine, Graduate School of Life Sciences, Kumamoto University, 1-1-1 Honjo, Chuo-ku, Kumamoto City, 860-8556 Japan

**Keywords:** Wild-type transthyretin-related amyloidosis cardiomyopathy, ^99m^Tc-pyrophosphate scintigraphy, SPECT/CT, Prognosis

## Abstract

**Purpose:**

Wild-type transthyretin-related amyloidosis cardiomyopathy (ATTRwt-CM) is an increasingly recognized cause of heart failure especially in elderly patients. The purpose of the present study was to determine retrospectively whether the quantitative indices of ^99m^Tc-pyrophosphate (PYP) SPECT/CT help to predict the prognosis of ATTRwt-CM patients when compared with other clinical parameters.

**Materials and methods:**

Sixty-eight patients with biopsy-proven ATTRwt-CM who underwent PYP SPECT/CT were enrolled. Baseline clinical characteristics, echocardiographic parameters, and qualitative and/or quantitative indices of planar and SPECT/CT imaging in PYP scintigraphy for each patient were included. For quantitative analysis of SPECT/CT, the accumulation ratio of PYP in the septum, posterior, anterior, lateral, and apex walls to the cavity pool was calculated as the septal wall-to-cavity ratio (Se/C), lateral wall-to-cavity ratio (La/C), anterior wall-to-cavity ratio (An/C), inferior wall-to-cavity ratio (In/C), and apical wall-to-cavity ratio (Ap/C), respectively. Endpoints for prognostic accuracy evaluation were cardiac death or hospitalization due to heart failure. Event-free survival rate was evaluated through Cox proportional hazards regression analysis, providing estimated hazard ratios (HRs) with 95% confidence intervals (CIs) and Kaplan–Meier curves.

**Results:**

High-sensitivity cardiac troponin T (hs-cTnT), La/C, age, interventricular septal thickness in diastole, and *E*/*e*′ ratio in the septal wall were significantly associated with event-free survival (*P* < 0.05). For a multivariable Cox proportional hazards analysis, hs-cTnT (HR 1.153; 95% CI 1.034–1.286; *P* < 0.01), La/C (HR 2.091; 95% CI 1.012–4.322; *P* = 0.046), and age (HR 1.116; 95% CI 1.007–1.238; *P* = 0.037) were significant independent prognostic factors.

**Conclusion:**

This study indicated that the quantitative indices of PYP SPECT/CT can help to predict the prognosis of ATTRwt-CM patients.

## Introduction

Wild-type transthyretin-related amyloidosis cardiomyopathy (ATTRwt-CM) is associated with heart failure in a considerable number of elderly patients [1]. An autopsy study revealed that at least 25% of individuals aged > 80 years had histological evidence of amyloid deposits in their cardiac tissue [2]. Another study reported that 13% of patients with heart failure with preserved ejection fraction (HFpEF) with left ventricular (LV) hypertrophy (> 12 mm) had been diagnosed with ATTRwt-CM [1].

Cardiac amyloidosis (CA) results from the progressive accumulation of amyloid protein in the myocardial interstitium and is associated with increased wall thickness, and it can cause diastolic and systolic dysfunctions; cardiac involvement greatly influences the prognosis outcome [3]. The gold standard for the definitive diagnosis of CA is endomyocardial biopsy coupled with either immunohistochemistry or, even more conclusively, mass spectroscopy [4].

Bone-avidity, phosphate-based isotopes, including ^99m^Tc-pyrophosphate (PYP) and ^99m^Tc-3,3-diphosphono-1,2-propanodiacarboxylic acid (DPD), show specific features for transthyretin-related amyloidosis (ATTR) deposits. International scientific literature agrees on the usefulness of bone-avid compounds for the accurate identification of transthyretin [5], and PYP scintigraphy can be beneficial in identifying amyloid deposition in the myocardium of patients with ATTR cardiomyopathy (ATTR-CM), and the scintigraphy constitutes a valuable method for the noninvasive diagnosis of ATTR-CM [6, 7].

For qualitative and quantitative evaluation of myocardial accumulation of PYP planar images in ATTR-CM patients, the Perugini grading system and heart-to-contralateral ratio (H/CL) are reported to have prognostic utility [8]. However, the benefits of qualitative and quantitative evaluation of PYP planar imaging or DPD scintigraphy in predicting accurate prognosis of patients with ATTR-CM remain controversial [9–11]. Although the diagnostic utility of ATTR-CM using PYP or ^99m^Tc-methylene diphosphonate (MDP) scintigraphy is said to be more useful for single-photon emission tomography (SPECT) than planar imaging [11–13], there are few studies on the actual efficacy of SPECT for predicting the prognosis of ATTR-CM patients [11]. Furthermore, there are no published studies on the usefulness of PYP single-photon emission tomography/computed tomography (SPECT/CT) when limited to only ATTRwt-CM patients. Therefore, the purpose of the present study was to determine whether quantitative indices of PYP SPECT/CT are useful for predicting the prognosis of cases limited to those diagnosed as ATTRwt-CM patients.

## Materials and methods

### Study population

Between November 2013 and November 2020, 568 patients underwent PYP SPECT/CT at our hospital. Of these, 148 patients had cardiac biopsy and 85 proved to be ATTRwt-CM cases. Of the 85 patients with ATTRwt-CM, 17 were excluded due to inadequate follow-up data. Ultimately, 68 patients were enrolled and their baseline demographic characteristics, co‐morbidities, laboratory results, echocardiographic data, imaging data, and pathological findings were recorded. Clinical examination was performed while each patient was in a clinically stable, non‐congestion condition.

This study was conducted in accordance with the principles outlined in the Declaration of Helsinki. The institutional review board of our institution approved the study and waived the necessity of obtaining informed consent.

### Diagnosis of wild‐type transthyretin amyloid cardiomyopathy

Diagnosis of amyloid deposition in the myocardium was based on Congo red staining and apple‐green birefringence with cross‐polarized light microscopy. To confirm the presence of transthyretin (TTR) in the amyloid, immunohistochemical staining was performed using antibodies that react with TTR. Wild-type transthyretin-related amyloidosis (ATTRwt) was diagnosed based on the absence of mutation in the TTR gene, which was ascertained through genetic testing, or the absence of a family history of amyloidosis in the elderly patients if genetic testing was not performed.

### Baseline clinical characteristics

Each patient’s baseline clinical characteristics were classified as follows: gender, age, body mass index (BMI), body surface area (BSA), history of hypertension, diabetes mellitus (DM), dyslipidemia (DL), chronic kidney disease (CKD), systolic blood pressure (SBP), diastolic blood pressure (DBP), hemoglobin (Hb), hemoglobin A1c (HbA1c), high-density lipoprotein cholesterol (HDL-cho), low-density lipoprotein cholesterol (LDL-cho), creatinine (Cr), estimated glomerular filtration rate (eGFR), C-reactive protein (CRP), brain natriuretic peptide (BNP), high-sensitivity cardiac troponin T (Hs-cTnT), New York Heart Association (NYHA) functional Class, and each indicator of echocardiography.

### Echocardiography

Echocardiography was performed using commercially available ultrasound equipment. Chamber size and wall thickness were measured in the transthoracic view. Echocardiographic findings included left ventricular ejection fraction (LVEF), left ventricular end diastolic volume (LVEDV), left ventricular end systolic volume (LVESV), interventricular septal thickness in diastole (IVSTd), posterior left ventricular wall thickness in diastole (PLVWd), and the ratio between early mitral inflow velocity and mitral annular early diastolic velocity (*E/eʹ*) in the septal and lateral wall (*E/eʹ* septal and *E/eʹ* lateral, respectively), which was determined by pulsed wave tissue Doppler imaging. Trans-mitral peak flow velocities were measured from mitral inflow velocities. LVEF was calculated using a modified Simpson’s method [14].

### PYP scintigraphy

PYP scintigraphy was performed using a GE Discovery 670 dual‐headed SPECT/CT camera (GE Healthcare, Waukesha, WI, USA). All patients were intravenously administered 555–740 MBq of PYP and planar and SPECT/CT imaging were performed 3 h later. Anterior and lateral planar images were acquired for 5 min each with a large field of view (FOV), using a 256 × 256 matrix with a 1.5 zoom factor, and the energy window was set at 140.5 keV (± 10%) with low‐energy, high‐resolution (LEHR) collimators. Following the planar imaging, SPECT imaging was obtained with LEHR collimators using a 10% energy window centered on the 140.5 keV photopeak in the main-energy window and a 5% energy window centered on the 120.0 keV photopeak in the sub-energy window for the dual-energy-window (DEW) scatter correction**.** SPECT data were acquired with step-and-shoot mode for 20 s per projection (30 projections/head; total of 180° of data). To enable CT-based attenuation correction (CTAC), non-contrast enhanced helical CT images (matrix, 512 × 512 pixels; slice thickness, 5 mm; and slice interval, 5 mm) were obtained with breath-holding in resting exhalation position. After registration of the SPECT and CT images, a CT-derived attenuation-coefficient map was created. Projections and reconstructed matrix size were 128 × 128 pixels, with a pixel size of 4.42 × 4.42 mm. The 3-dimensional ordered subset expectation maximization (3D-OSEM) iterative reconstruction (2 iterations, 10 subsets) was done both with CTAC and DEW scatter correction. 3D-OSEM images were also filtered after reconstruction with a Butterworth filter (cutoff 0.48 cycles/cm, power factor = 10). Reconstruction of SPECT images was performed on the Xeleris workstation (GE Healthcare, Waukesha, WI, USA).

**Fig. 1:**
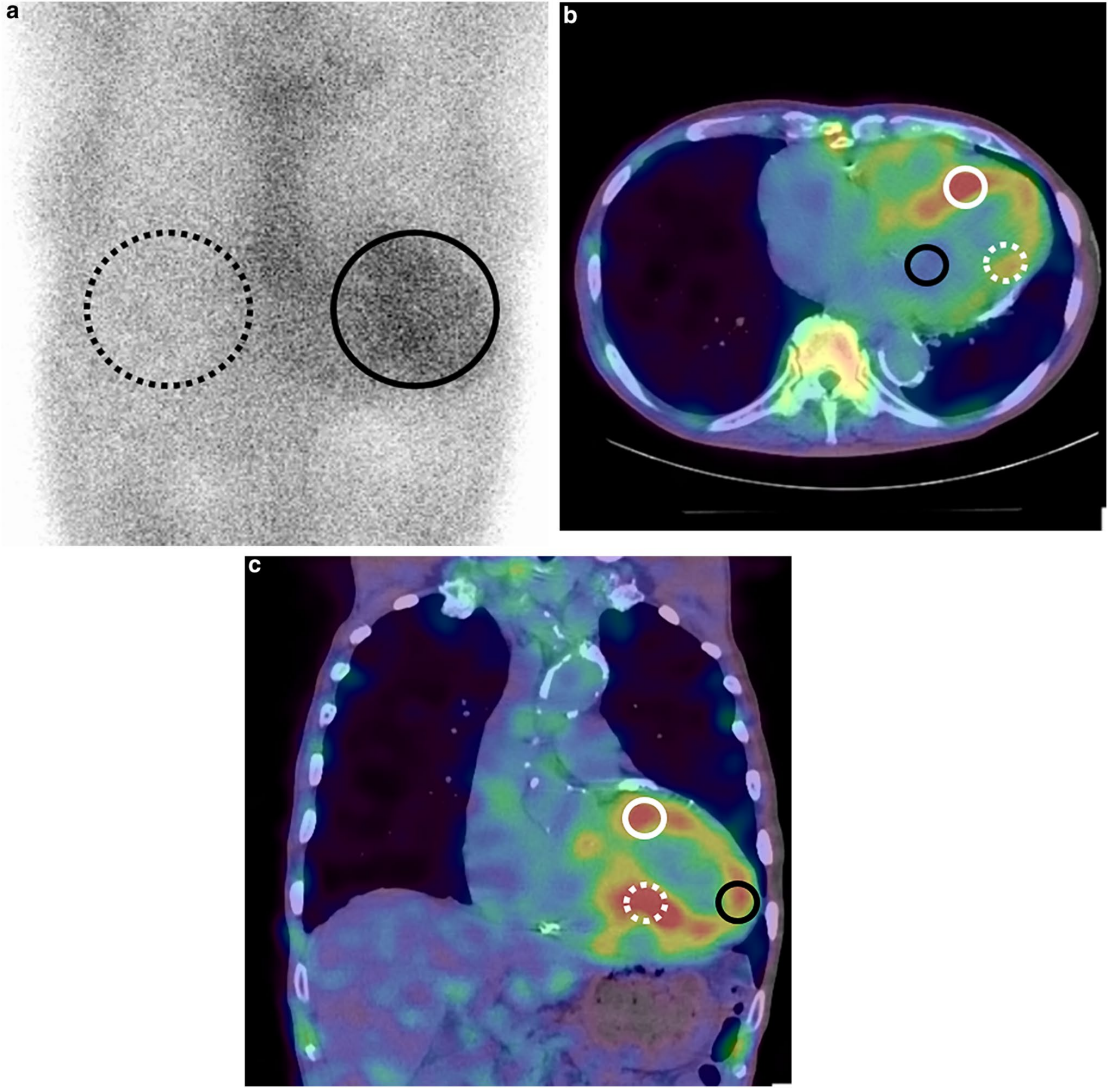
ROIs setting in PYP planar and SPECT/CT images. On planar image (**A**), a circular ROI was put on the heart (black circle) and mirroring it to the contralateral chest (black dashed circle) to calculate H/CL. On SPECT/CT fusion images (**B**, **C**), a circular ROI was set at the maximum count area of PYP accumulation in five regions of the left ventricular myocardium and at the minimum count area in the left ventricular cavity (**B**; black circle) on axial image. The ROIs were set for the septal (**B**; white circle) and lateral regions (**B**; white dashed circle) on the axial image and apical (**C**; black circle), anterior (**C**; white circle), and inferior walls (**C**; white dashed circles) on the coronal image (**C**)

For quantitative analysis in both planar and SPECT images, region-of-interest (ROI) measurements were performed in consensus with two experienced nuclear imaging specialists blinded to all patient data. For the analysis of planar images, a circular ROI was drawn over the heart, which was copied and mirrored to the contralateral chest to normalize background uptake. Mean total heart ROI counts were measured and corrected for contralateral chest ROI counts by calculating a heart-to-contralateral (H/CL) ratio [15]. In the ROI analysis of SPECT images, the slice with the widest depiction of the left ventricular cavity and the adjacent slice were selected with reference to SPECT/CT fusion images. A 1 cm^2^ circular ROI was placed at the maximum uptake area of the septal and lateral walls on the axial SPECT/CT image and of the anterior, inferior and apical walls on the coronal SPECT/CT image (Fig. 1). A 1 cm^2^ circular ROI was also put at the minimal uptake area of the cardiac cavity blood pool on the axial or coronal image calculate the septal wall-to-cavity ratio (Se/C), lateral wall-to-cavity ratio (La/C), anterior wall-to-cavity ratio (An/C), inferior wall-to-cavity ratio (In/C), and apical wall-to-cavity ratio (Ap/C).

### Statistical analysis

The endpoint for prognostic assessment was cardiac death or hospitalization due to heart failure. For each patient in the event group, an event date was set as the first occurrence of one of the events during the follow-up period. Baseline dates were the same as the date when PYP scintigraphy was performed. Associations between the baseline clinical characteristics and the prognosis were analyzed.

Parameters of continuous variables were expressed as mean ± standard deviation (SD), whereas categorical values were presented as numbers (percentages). Continuous variables were compared in two groups (event-free group vs. event group) with the student *t* test. Categorical variables were compared using the Chi-square test. Event-free survival was evaluated with Cox proportional hazards regression analysis, providing estimated hazard ratios (HRs) with 95% confidence intervals (CIs) and Kaplan–Meier curves. All variables were first explored with univariate Cox regression analysis. The variables that were statistically significant predictors of prognosis on simple Cox regression analysis were entered into a multivariable Cox proportional hazards analysis to determine which covariates were independent predictors of a prognosis. Patients were stratified according to indicators with significant differences and patient prognosis was compared using the log-rank test. The optimal cutoff value of each parameter was determined as a value to maximize the Youden Index. A two‐tailed value of *P* < 0.05 was considered statistically significant. All statistical analyses were performed using SPSS software (version 24.0.0, SPSS Inc., Chicago, IL, USA).

## Results

Baseline characteristics of all patients are shown in Table 1. Sixty-one (90%) patients were male, and mean age at baseline was 75 years. The median follow-up period was 667 days (range 27–2364 days). During the follow-up period, event occurrence was observed in 29 (43%) patients: 7 patients died and 22 were hospitalized due to heart failure.

**Table 1:** Baseline characteristics of 68 patients

Characteristics	Total *n* = 68 (± SD, %)	Event-free group *n* = 46 (± SD, %)	Event group *n* = 22 (± SD, %)	*P* value
Age (years)	75 ± 6	74 ± 6	79 ± 5	0.007
BMI (kg/m^2^)	24 ± 3	24 ± 3	23 ± 3	0.449
BSA (m^2^)	1.65 ± 0.14	1.67 ± 0.14	1.61 ± 0.13	0.094
SBP (mmHg)	117 ± 19	116 ± 18	121 ± 20	0.282
DBP (mmHg)	72 ± 14	72 ± 15	70 ± 12	0.599
Hb (g/dL)	13.9 ± 1.9	14.1 ± 1.9	13.5 ± 19	0.236
HbA1c (%)	6.2 ± 0.7	6.2 ± 0.7	6.2 ± 0.6	0.789
Cr (mg/dL)	1 ± 0.2	1 ± 0.2	1 ± 0.3	0.709
eGFR (mL/min)	55 ± 12	56 ± 12	53 ± 12	0.336
LDL-cho (mg/dL)	101 ± 29	103 ± 29	96 ± 28	0.342
HDL-cho (mg/dL)	58 ± 15	59 ± 14	57 ± 16	0.584
CRP (mg/dL)	0.19 ± 0.27	0.2 ± 0.27	0.17 ± 0.28	0.711
BNP (pg/mL)	276 ± 169	262 ± 176	306 ± 153	0.322
Hs-cTnT (ng/mL)	0.065 ± 0.043	0.058 ± 0.038	0.079 ± 0.52	0.063
Gender (male)	61 (90)	43 (93)	18 (82)	0.214
HT	37 (54)	24 (52)	13 (59)	0.599
DM	19 (28)	14 (30)	5 (23)	0.515
DL	26 (38)	18 (39)	8 (36)	0.829
CKD	23 (34)	14 (30)	9 (41)	0.401
Smoking	36 (53)	29 (63)	7 (32)	0.015
NYHA class				0.486
1	14 (21)	9 (20)	5 (24)	
2	36 (54)	23 (50)	13 (62)	
3	16 (24)	14 (30)	2 (10)	
4	1 (1)	0 (0)	1 (5)	
Echocardiogram parameters				
LVESV (mL)	37 ± 15	37 ± 17	37 ± 13	0.949
LVEDV (mL)	74 ± 21	75 ± 22	72 ± 19	0.685
LVEF (%)	51 ± 10	52 ± 11	50 ± 8	0.354
IVSTd (mm)	15.8 ± 2.5	15.5 ± 2.3	16.4 ± 2.8	0.183
PLVWd (mm)	16.3 ± 2.7	16.2 ± 2.5	16.5 ± 3.1	0.670
*E/eʹ* septal	21.2 ± 6.9	20.4 ± 6.6	23.1 ± 7.3	0.136
*E/eʹ* lateral	15.7 ± 5.7	15.2 ± 5.5	17.6 ± 6.3	0.239
Scintigraphic parameters				
H/CL	1.79 ± 0.32	1.83 ± 0.33	1.7 ± 0.26	0.107
Se/C	3.02 ± 0.82	2.96 ± 0.84	3.14 ± 0.79	0.395
An/C	2.88 ± 0.73	2.78 ± 0.72	3.1 ± 0.7	0.092
Ap/C	2.62 ± 0.67	2.52 ± 0.71	2.81 ± 0.55	0.104
In/C	2.62 ± 0.61	2.52 ± 0.62	2.83 ± 0.53	0.050
La/C	2.19 ± 0.59	2.1 ± 0.59	2.39 ± 0.55	0.058

*BMI* body mass index, *BSA* body surface area, *SBP* systolic blood pressure, *DBP* diastolic blood pressure, Hb hemoglobin, *HbA1c* hemoglobin A1c, *Cr* creatinine, *eGFR* estimated glomerular filtration rate, *LDL-cho* low-density lipoprotein cholesterol, *HDL-cho* high-density lipoprotein cholesterol, *CRP* C-reactive protein, *BNP* brain natriuretic peptide, *Hs-cTnT* high-sensitivity cardiac troponin T, *HT* hypertension, *DM* diabetes mellitus, *DL* dyslipidemia, *CKD* chronic kidney disease, *NYHA* New York Heart Association, *LVESV* left ventricular end systolic volume, *LVEDV* left ventricular end diastolic volume, *LVEF* left ventricular ejection fraction, *IVSTd* interventricular septal thickness in diastole, *PLVWd* posterior left ventricular wall thickness in diastole, *H/CL* heart-to-contralateral ratio, *Se/C* septal wall-to-cavity ratio, *An/C* anterior wall-to-cavity ratio, *Ap/C* apical wall-to-cavity ratio, *In/C* inferior wall-to-cavity ratio, *La/C* lateral wall-to-cavity ratio

Univariate Cox regression analysis for the baseline characteristics, echocardiographic parameters, and metrics of planar and SPECT/CT imaging in PYP scintigraphy is shown in Table 2. Hs-cTnT, La/C, age, IVSTd, and *E/eʹ* septal were significantly associated with event-free survival (*P* < 0.05); the HR was 1.137 (95% CI 1.055–1.225) for hs-cTnT, 2.297 (95% CI 1.146–4.601) for La/C, 1.085 (95% CI 1.001–1.175) for age, 1.177 (95% CI 1.009–1.373) for IVSTd, and 1.064 (95% CI 1.002–1.130) for *E/eʹ* septal. In the multivariable Cox proportional hazards analysis (Table 3), a significant, independent factor of prognosis was found for hs-cTnT (HR 1.153; 95% CI 1.034–1.286; *P* < 0.01), La/C (HR 2.091; 95% CI 1.012–4.322; *P* = 0.046), and age (HR 1.116; 95% CI 1.007–1.238; *P* = 0.037) (Table 3).

**Table 2:** Univariate analysis for 68 ATTRwt-CM patients

Predictive factors	Hazard ratio (95% CI)	*P* value
Gender (male)	1.987 (0.658–5.997)	0.223
Age (years)	1.085 (1.001–1.175)	0.046
BMI (kg/m^2^)	0.942 (0.814–1.089)	0.417
BSA (m^2^)	0.059 (0.003–1.145)	0.061
HT	1.252 (0.518–3.024)	0.617
DM	0.918 (0.330–2.551)	0.869
DL	1.158 (0.475–2.824)	0.748
CKD	1.541 (0.643–3.694)	0.333
Smoking	0.522 (0.210–1.296)	0.161
SBP (mmHg)	1.024 (0.994–1.055)	0.117
DBP (mmHg)	0.966 (0.924–1.010)	0.125
HbA1c (%)	0.983 (0.562–1.721)	0.952
HDL-cho (mg/dL)	0.985 (0.951–1.019)	0.371
LDL-cho (mg/dL)	0.996 (0.979–1.013)	0.647
Cr (mg/dL)	2.045 (0.311–13.428)	0.456
eGFR (mL/min)	0.978 (0.943–1.014)	0.228
CRP (mg/dL)	1.122 (0.211–5.968)	0.893
Hb (g/dL)	0.859 (0.665–1.109)	0.243
BNP (pg/mL)	1.002 (1.000–1.005)	0.062
Hs-cTnT (ng/mL)	1.137 (1.055–1.225)	0.001
NYHA class	0.874 (0.446–1.712)	0.694
LVEF (%)	0.972 (0.933–1.013)	0.182
IVSTd (mm)	1.177 (1.009–1.373)	0.038
PLVWd (mm)	1.113 (0.944–1.312)	0.204
*E/eʹ* septal	1.064 (1.002–1.130)	0.044
*E/eʹ* lateral	1.077 (0.969–1.196)	0.167
LVESV	1.002 (0.973–1.032)	0.889
LVEDV	0.991 (0.970–1.013)	0.435
H/CL	0.495 (0.110–2.219)	0.358
Se/C	1.218 (0.781–1.898)	0.385
An/C	1.477 (0.852–2.561)	0.165
Ap/C	1.631 (0.847–3.143)	0.144
In/C	1.497 (0.847–2.647)	0.165
La/C	2.297 (1.146–4.601)	0.019

*BMI* body mass index, *BSA* body surface area, *HT* hypertension, *DM* diabetes mellitus, *DL* dyslipidemia, *CKD* chronic kidney disease, *SBP* systolic blood pressure, *DBP* diastolic blood pressure, *HbA1c* hemoglobin A1c, *HDL-cho* high-density lipoprotein cholesterol, *LDL-cho* low-density lipoprotein cholesterol, *Cr* creatinine, *eGFR* estimated glomerular filtration rate, *CRP* C-reactive protein, *Hb* hemoglobin, *BNP* brain natriuretic peptide, *Hs-cTnT* high-sensitivity cardiac troponin T, *NYHA* New York Heart Association, *LVEF* left ventricular ejection fraction, *IVSTd* interventricular septal thickness in diastole, *PLVWd* posterior left ventricular wall thickness in diastole, *LVESV* left ventricular end systolic volume, *LVEDV* left ventricular end diastolic volume, *H/CL* heart-to-contralateral ratio, *Se/C* septal wall-to-cavity ratio, *An/C* anterior wall-to-cavity ratio, *Ap/C* apical wall-to-cavity ratio, *In/C* inferior wall-to-cavity ratio, *La/C* lateral wall-to-cavity ratio

**Table 3:** Multivariate analysis for 68 ATTRwt-CM patients

Predictive factors	Hazard ratio (95% CI)	*P* value
Hs-cTnT	1.153 (1.034–1.286)	0.01
La/C	2.091 (1.012–4.322)	0.046
Age	1.116 (1.007–1.238)	0.037
IVSTd	1.032 (0.867–1.228)	0.725
*E/eʹ* septal	1.022 (0.955–1.095)	0.527

*CI* confidence intervals, *Hs-cTnT* high-sensitivity cardiac troponin T, *La/C* lateral wall-to-cavity ratio, *IVSTd* interventricular septal thickness in diastole

**Fig. 2:**
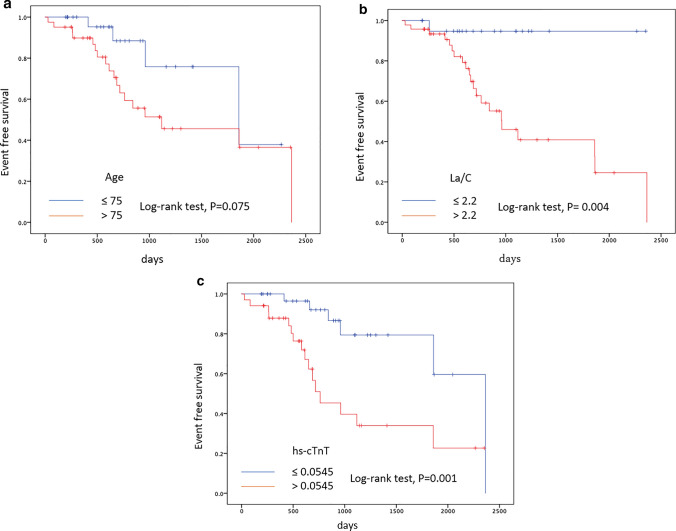
Kaplan–Meier curves showing event-free survival probabilities in patients with ATTRwt-CM. **A** Stratified by age > 75 years vs. ≤ 75 years (log-rank test, *P* = 0.075). **B** Stratified by La/C ≥ 2.2 vs. La/C < 2.2 (log-rank test, *P* = 0.004). **C** Stratified by hs-cTnT ≥ 0.0546 vs. hs-cTnT < 0.0546 (log-rank test, *P* = 0.001)

Kaplan–Meier analysis showed that La/C > 2.2 and hs-cTnT > 0.0545 were found to be significantly associated with event-free survival (*P* < 0.004 and < 0.001, respectively) (Fig. 2). Patients with La/C > 2.2 had a median survival of 961 days (95% CI 599–1323 days), patients with hs-cTnT > 0.0545 had a median survival of 762 days (95% CI 414–1109 days), and patients aged > 75 had a median survival of 1117 days (95% CI 79–2154 days).

## Discussion

**Fig. 3:**
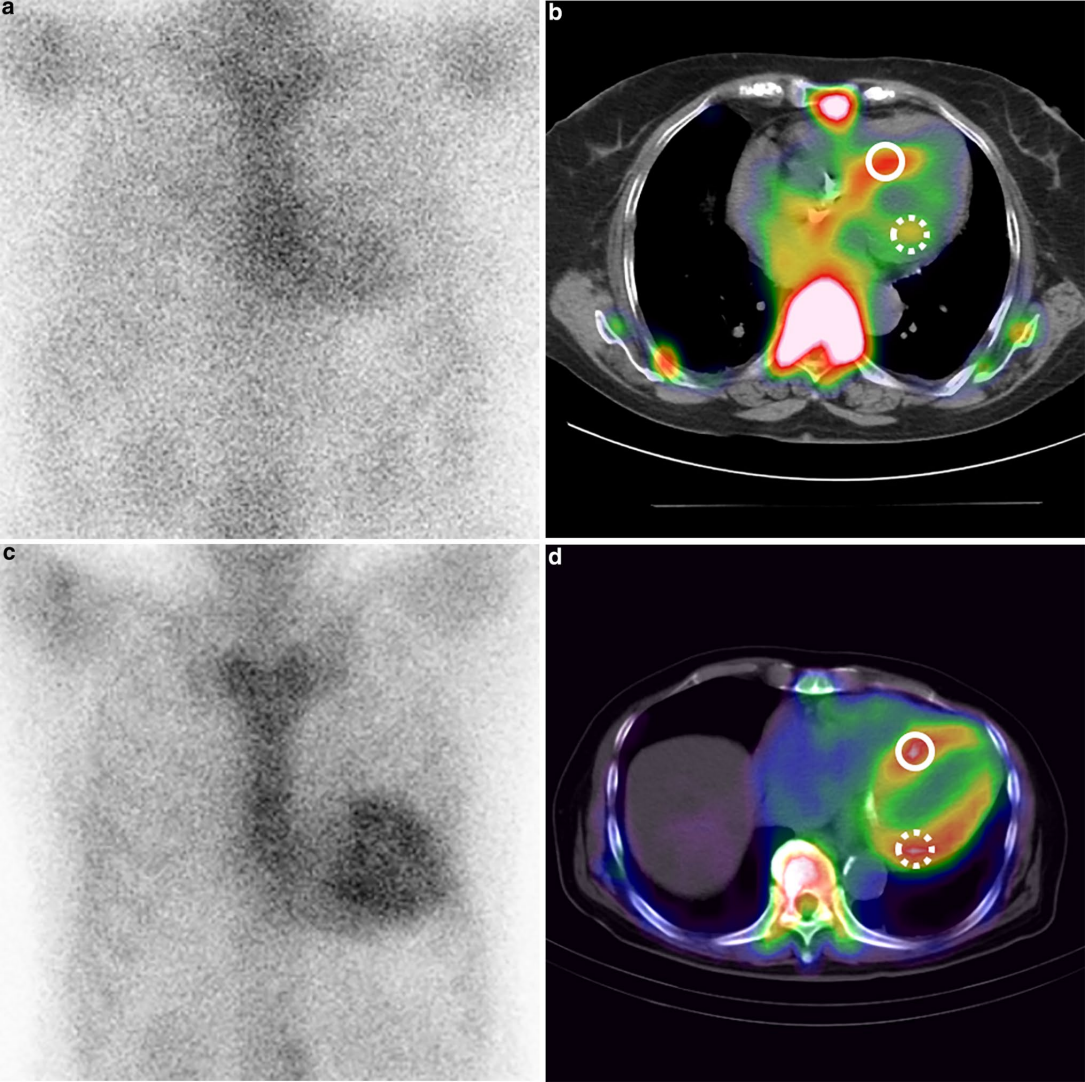
PYP planar and SPECT/CT images of two ATTRwt-CM patients. Circles indicate the region of interest for the highest PYP accumulation in the septal and lateral walls of the left ventricle. **A**, **B** An 86-year-old female patient with ATTRwt-CM had an hs-cTnT level of 0.027 ng/mL and an event-free survival of 1441 days. **A** The planar image shows mild PYP accumulation in the left ventricular myocardium with an H/CL of 1.41. **B** In SPECT/CT fusion image, PYP accumulation is obviously lower in the left ventricular lateral wall (dashed circle) with a La/C of 1.51 than that in the septal wall (circle) with a Se/C of 2.01. **C**, **D** A 79-year-old male patient with ATTRwt-CM had an hs-cTnT level of 0.0855 ng/mL and was hospitalized for heart failure on day 588 and died on day 1880. **C** The planar image shows diffuse PYP high accumulation in the left ventricular myocardium with an H/CL of 1.99. **D** On SPECT/CT fusion image, the PYP accumulation is slightly higher in the lateral wall (dashed circle) with a La/C of 2.80 than the septal wall (circle) with a Se/C of 2.52

This study found that, among the metrics of myocardial uptake on PYP planar and SPECT/CT images, La/C was a significant independent factor for predicting the prognosis of ATTRwt-CM patients. Two typical cases showing this are presented in Fig. 3. In predicting the prognosis of patients with ATTR-CM, the usefulness of the qualitative and quantitative evaluation of the PYP planar or DPD scintigraphy is controversial [9–11] and there are hardly any reports on the usefulness of quantitative SPECT evaluation [11]. As far as we know, this report is the first study to show that quantitative metrics with PYP SPECT/CT when limited to ATTRwt-CM cases can help to predict the prognosis of such patients.

In our study, the localization of PYP accumulation in the myocardium, especially in the lateral wall, predicted the prognosis in patients with ATTRwt-CM. We do not know the exact reason, but we assume as follows. Grigoratos et al. [16] showed that tracer accumulation in the early stages of the disease was higher in the septal wall than in the other regions, but in the later stages, the accumulation was almost uniform throughout the left ventricular myocardium. Their data suggest that amyloid deposition may progress from the septal wall to the other regions including the lateral wall. In our study, the difference in the tracer accumulation between the event-free group and the event group was much weaker in the septal wall than in the lateral wall. Based on these data, we speculate: Amyloid deposits begin in the septal wall in the early stages of the disease and then gradually spread throughout the left ventricular myocardium. In addition, accumulation in the lateral wall, which is the furthest part of the septum, occurs most late and increases to levels similar to accumulation in the septum and other regions. That is, if accumulation in the lateral wall progresses to the similar extent as in other regions, especially the septum, cardiac dysfunction can be exacerbated as a result of diffuse amyloid deposits throughout the myocardium.

Castano et al. [9] showed that ATTR cardiac amyloidosis patients of having an H/CL ratio of 1.6 or greater predicted worse survival. However, in our study, we could not find a significant difference in H/CL ratio between the event-free group and the event group. The subjects of the study by Castano et al. [9] included CM patients with hereditary ATTR, wild-type ATTR, and unknown genotype ATTR. In contrast, the subjects of our study were only ATTRwt-CM patients. Our study showed the event group was older than the event-free group, but the correlation coefficient between the H/CL ratio and age was -0.192, which was a negative correlation. This is probably one of the reasons why the H/CL ratio negated an association with prognosis. Although the natural history of ATTRwt-CM patients has not been clarified, myocardial deposition of amyloid might vary depending on the time of onset. We speculate as follows. In late-onset cases, the myocardium may have slow amyloid deposits and relatively low PYP accumulation, whereas in early-onset cases, it may have fast amyloid deposits and relatively high PYP accumulation. Another possible reason is that H/CL ratio can make cardiac pool images false positive [13]. This can lead to a false assessment of tracer accumulation on the myocardial wall, unlike the assessment with SPECT/CT fusion images.

In this study, hs-cTnT and also age were significant independent factors for predicting prognosis in ATTRwt-CM patients. Some prognostic indicators for ATTR-CM patients have been previously reported. A staging system with a combination of NT‐proBNP and serum hs‐cTnT levels or estimated glomerular filtration rate was proposed for predicting the survival period of ATTR‐CM patients [17, 18]*.* In addition, Kreusser et al. reported that hs-TnT, QRS duration, and N-terminal pro-brain natriuretic peptide (NT-pro BNP) were the best predictors for all causes of mortality in ATTRwt-CM patients with advanced heart failure due to amyloid cardiomyopathy [19]. Ochi et al. showed that low serum albumin, elevated hs-cTnT, and reduced LVEF were associated with a worse prognosis in Japanese patients with ATTRwt-CM [20]. Yamada et al. showed that age and serum sodium levels were suggested as factors contributing to the mortality [21]*.* In our study, BNP and albumin levels, and LVEF were not statistically significant indicators as prognostic factors.

Although several SPECT quantification methods have been presented, this study used the accumulated ratio of the myocardial wall to the left ventricular cavity as the quantitative indices of PYP SPECT/CT. A standardized uptake value (SUV) can be used as a quantitative index of PYP SPECT/CT [22, 23]. However, measurement of the SUV requires data such as body weight, dose of radiopharmaceutical drug, time from administration to imaging, and also processing software is needed [24], and the equipment that can perform the measuring is not in widespread use. In addition, PYP accumulates not only in the myocardium but also in various other organs, and the degree of myocardial accumulation depends on the degree of accumulation in other organs. Therefore, the exact measurement of SUV for PYP accumulation may not necessarily directly reflect amyloid deposits in the myocardium. The polar map method may be also used as a quantitative index of PYP SPECT/CT [11, 16]. However, this method makes it difficult to properly delineate the contours of the left ventricular myocardium in cases with poor PYP accumulation. The strategy is suitable for assessing the distribution of PYP accumulation, but not for its quantitative assessment. Compared with the above methods, the assessment method used in this research is clinically practical without the need for special software, which is unavailable in many medical institutions.

Our study has some limitations. First, the number of patients was relatively small. Only patients with ATTRwt-CM confirmed by cardiac biopsy were included. Second, cardiac death or hospitalization due to heart failure became the endpoints for the prognostic assessment as the duration of the research was relatively short. A longer period is required to clearly assess the survival rate of ATTRwt-CM patients. Finally, late gadolinium enhancement (LGE) and extracellular volume (ECV) data from the MR imaging were not used in this study. Further study is being planned to compare the degree and distribution of PYP myocardial accumulation with ECV and LGE data from MRI.

## Conclusions

This study showed that La/C, hs-cTnT, and age were significantly independent prognostic factors in ATTRwt-CM patients. Among the metrics of myocardial uptake on PYP planar and SPECT/CT images, La/C can be a useful biomarker for predicting the prognosis of such patients.
